# Patients’ attitudes and perceptions towards treatment of hypothyroidism in general practice: an in-depth qualitative interview study

**DOI:** 10.3399/bjgpopen17X100977

**Published:** 2017-06-28

**Authors:** Rosie Dew, Kathryn King, Onyebuchi E Okosieme, Simon Pearce, Gemma Donovan, Peter Taylor, Graham Leese, Janis Hickey, Salman Razvi, Colin Dayan, Scott Wilkes

**Affiliations:** 1 Research Associate, Faculty of Health Sciences and Wellbeing, University of Sunderland, Sunderland, UK; 2 Principle Lecturer, Faculty of Sciences and Wellbeing, University of Sunderland, Sunderland, UK; 3 Consultant Endocrinologist and Honorary Lecturer, Diabetes and Endocrinology Department, Prince Charles Hospital, Cwm Taf Health Board, Cardiff, UK; 4 Professor of Endocrinology, Institute of Human Genetics, Newcastle University, Newcastle, UK; 5 Academic Pharmacist Practitioner, Faculty of Health Sciences and Wellbeing, University of Sunderland, Sunderland, UK; 6 Welsh Clinical Academic Trainee, Institute of Molecular and Experimental Medicine, Cardiff University School of Medicine, Cardiff, UK; 7 Consultant and Professor in Diabetes and Endocrinology, University of Dundee, Ninewells Hospital and Medical School, Dundee, UK; 8 Founder and Director, British Thyroid Foundation, Harrogate, UK; 9 Consultant Endocrinologist and Senior Lecturer, Institute of Human Genetics, Newcastle University, Newcastle, UK; 10 Professor of Clinical Diabetes and MetabolismInstitute of Molecular and Experimental Medicine, Institute of Molecular and Experimental Medicine, Cardiff University School of Medicine, Cardiff, UK; 11 Professor of General Practice and Primary Care, Faculty of Health Sciences and Wellbeing, University of Sunderland, Sunderland, UK

**Keywords:** general practice, levothyroxine, hypothyroidism, patient attitudes, TSH

## Abstract

**Background:**

Suboptimal thyroid hormone replacement is common in patients with hypothyroidism and the behavioural factors underlying this are poorly understood.

**Aim:**

To explore the attitudes and perceptions of patients to thyroid hormone replacement therapy.

**Design & setting:**

An in-depth qualitative interview study with patients with hypothyroidism residing in Northumberland, and Tyne and Wear, UK.

**Method:**

Twenty-seven patients participated, of which 15 patients had thyroid stimulating hormone (TSH) levels within the reference range (0.4–4.0 mU/L) and 12 patients had TSH levels outside the reference range. A grounded theory approach was used to explore and develop emerging themes, which were mapped to the health belief model (HBM).

**Results:**

Patients generally had a low understanding of their condition or of the consequences of suboptimal thyroid hormone replacement. Patients that had experienced hypothyroid symptoms at initial diagnosis had a better perception of disease susceptibility, and this was reflected in excellent adherence to levothyroxine in this group of patients. The main benefits of optimal thyroid replacement were improved wellbeing and performance. However, patients who remained unwell despite a normal serum TSH level felt that their normal result presented a barrier to further evaluation of their symptoms by their GP.

**Conclusion:**

Educating patients with hypothyroidism regarding the consequences of inadequate thyroid hormone replacement may reduce barriers and improve treatment outcomes. An over-reliance on TSH as a sole marker of wellbeing reduced opportunities for clinicians to address patient symptoms. Evaluating symptoms in combination with biochemical indices, may lead to better patient outcomes than relying on laboratory tests alone.

## How this fits in

There is limited information on the behavioural factors that influence disease outcomes in patients with hypothyroidism. This qualitative study of patients’ attitudes and perceptions shows that patients generally adhered well to treatment but were often unable to obtain clear, reliable information about their disease or its treatment. A TSH concentration within the reference range was perceived by most patients to be an unsatisfactory measure of wellbeing that often precluded meaningful interaction with their practitioners. Outcomes may be improved by the provision of better quality information, together with a clinical approach that addresses patients’ symptoms and concerns, alongside their biochemical indices of thyroid function.

## Introduction

Hypothyroidism is a chronic condition affecting 2–5% of the population.^1^ About 3% of the UK population receive levothyroxine therapy^2^ with the aim of treatment being to achieve patient wellbeing and restore serum thyroid stimulating hormone (TSH) levels to within the reference range.^3^ Levothyroxine is the third most dispensed drug in England with 29.7 million items dispensed in 2015 alone.^4^ After individual dose adjustment levothyroxine is generally well-tolerated and most patients continue lifelong therapy without complications.^5^

Suboptimal replacement as shown by abnormal TSH levels, however, has remained problematic for over 20 years,^6,7^ despite increasing regularity in the biochemical monitoring of patients.^8–10^ Patients with suboptimal replacement may have an increased risk of cardiovascular events and fractures.^11–13^ Furthermore, women of childbearing age established on levothyroxine often have out-of-range TSH levels at the time of conception, increasing the risk of adverse pregnancy outcomes.^14^ Reduced quality of life is common among patients with hypothyroidism, particularly relating to energy, motivation, physical capabilities, physical appearance, and weight gain.^15,16^ In addition, there are substantial healthcare resource implications of having abnormal TSH levels including repeat laboratory testing, levothyroxine dose adjustments, and medicines wastage.

Potential causes of inadequate thyroid hormone replacement include medication adherence^17,18^ as well as biochemical,^19^ pharmacogenomic,^20–23^ and medication formulation factors.^24^ To date, most guidance on managing thyroid hormone replacement has focused on physiological and pharmacological factors.^3,25^ However, there is little information on behavioural factors even though patient perception and attitudes towards their illness play a pivotal role in improving chronic disease outcomes.^26^ To begin to address this problem this study explored the behavioural factors that may contribute to suboptimal treatment in patients with hypothyroidism.

## Method

### Design

This study used in-depth qualitative interviews with patients with hypothyroidism to explore their experiences, attitudes, and perceptions towards their treatment. A grounded approach was used to develop emerging themes.^27^ The theoretical underpinning of the project was provided by the HBM^28^ which has two main components; perceived threat and behavioural evaluation aspects.^29^ The HBM enabled mapping of themes to give an overall explanation of patient’s ability and readiness to influence the control of their disease.

### Participants

The National Institute for Health Research (NIHR) Clinical Research Network North East and North Cumbria approached primary care practices in Northumberland and Tyne and Wear and nine agreed to take part. On average, each GP practice sent 30 study packs containing an invitation letter and information sheet to patients with overt hypothyroidism established on levothyroxine for ≥12 months. Around three patients per GP surgery chose to take part, while two patients approached the researcher after hearing about the study during a presentation. Twenty-seven interviews were conducted, of which 15 patients had TSH levels within the reference range (0.4–4.0 mU/L)^3^ and 12 had TSH levels outside the reference range. Participant characteristics are shown in Table 1.

**Table 1:** Patient demographics

Characteristic		Patients with TSH within reference range *N *= 15, *n* (%)	Patients with TSH lower than reference range *N *= 7, *n* (%)	Patients with TSH higher than reference range *N *= 5, *n* (%)
**Sex**	Female	11 (73)	4 (57)	3 (60)
	Male	4 (27)	3 (43)	2 (40)
**Location**	Rural	6 (40)	6 (86)	1 (20)
	Urban	9 (60)	1 (14)	4 (80)
**Age, years**	30–39	1 (7)	1 (14)	2 (40)
	40–49	1 (7)	1 (14)	1 (20)
	50–59	0 (0)	0 (0)	1 (20)
	60–69	8 (53)	3 (43)	1 (20)
	70–79	3 (20)	1 (14)	0 (0)
	80–89	2 (13)	1 (14)	0 (0)
**Years with hypothyroidism**	≤5	2 (13)	1 (14)	3 (60)
	6–10	2 (13)	4 (57)	0 (0)
	11–20	5 (33)	1 (14)	2 (40)
	>20	6 (40)	1 (14)	0 (0)
**Other relevant health conditions**	Coeliac disease	0 (0)	0 (0)	0 (0)
	Pernicious anaemia	2 (13)	0 (0)	0 (0)
	Gastrointestinal (gallstones/diverticulitis/irritable bowel syndrome/twisted bowel/constipation)	4 (27)	2 (29)	2 (40)
	Type 2 diabetes	3 (20)	0 (0)	0 (0)
**Other relevant medications**	Calcium-containing drugs	3 (20)	0 (0)	1 (20)
	Iron-containing drugs	0 (0)	1 (14)	1 (20)
	Proton pump inhibitors	3 (20)	1 (14)	1 (20)
**BMI**	<20	1 (7)	0	0
	20–25	5 (33)	1 (14)	2 (40)
	26–30	5 (33)	5 (71)	2 (40)
	31–35	3 (20)	1 (14)	1 (20)
	36–40	1 (7)	0	0
**Daily levothyroxine dose, µg**	25	1 (7)	0	0
	50	1 (7)	0	0
	75	2 (13)	0	0
	100	3 (20)	2 (29)	2 (40)
	125	3 (20)	2 (29)	3 (60)
	150	2 (13)	0	0
	175	0 (0)	0	0
	200	0 (0)	0	0
	225	0 (0)	1 (14)	0
	100/125 alternate day dosing	1 (7)	1 (14)	0
	100/75 alternate day dosing	0 (0)	1 (14)	0
	100 Monday–Friday, 75 Saturday–﻿Sunday	2 (13)	0	0

Interviews were conducted by the same researcher. An initial semistructured topic guide was used to explore questions related to the study and this was revised as the study progressed (Box 1). Interviews lasted around 45 minutes with only the researcher and patient present, except on two occasions when family members were present and contributed. Patients were given a £10 voucher for their participation. All interviews were audiorecorded, transcribed verbatim, and anonymised. The majority of interviews took place in the patient’s home with the exception of four patients who preferred their GP surgery, two patients the University of Sunderland, and one patient their place of work. Transcripts were returned to participants for comments and no patients asked for any changes.

**Box 1.** Initial topic guide for patient interviews

What do you think about the control of your hypothyroidism? Are you well controlled or poorly controlled? How often do you get your thyroid blood tests checked? How often do you feel you should have your blood test checked? How do you get your results? Are you aware of how well/poorly you are controlled? What do you understand about how your thyroid is controlled? Are you aware of any consequences of poor control? What are the symptoms of being out of control? How much of your life do you feel well controlled? Do you ever forget to take your tablets? If so what do you do? What else do you think may influence the control of your hypothyroidism?

### Sampling

Purposive sampling was used to initially identify participants from both rural and urban areas, and large and small GP practices, resulting in a maximum variation sample.^27^ Theoretical sampling was then used to test emerging categories and themes which proceeded until data saturation was achieved.^30^

### Analysis

**Figure 1.**
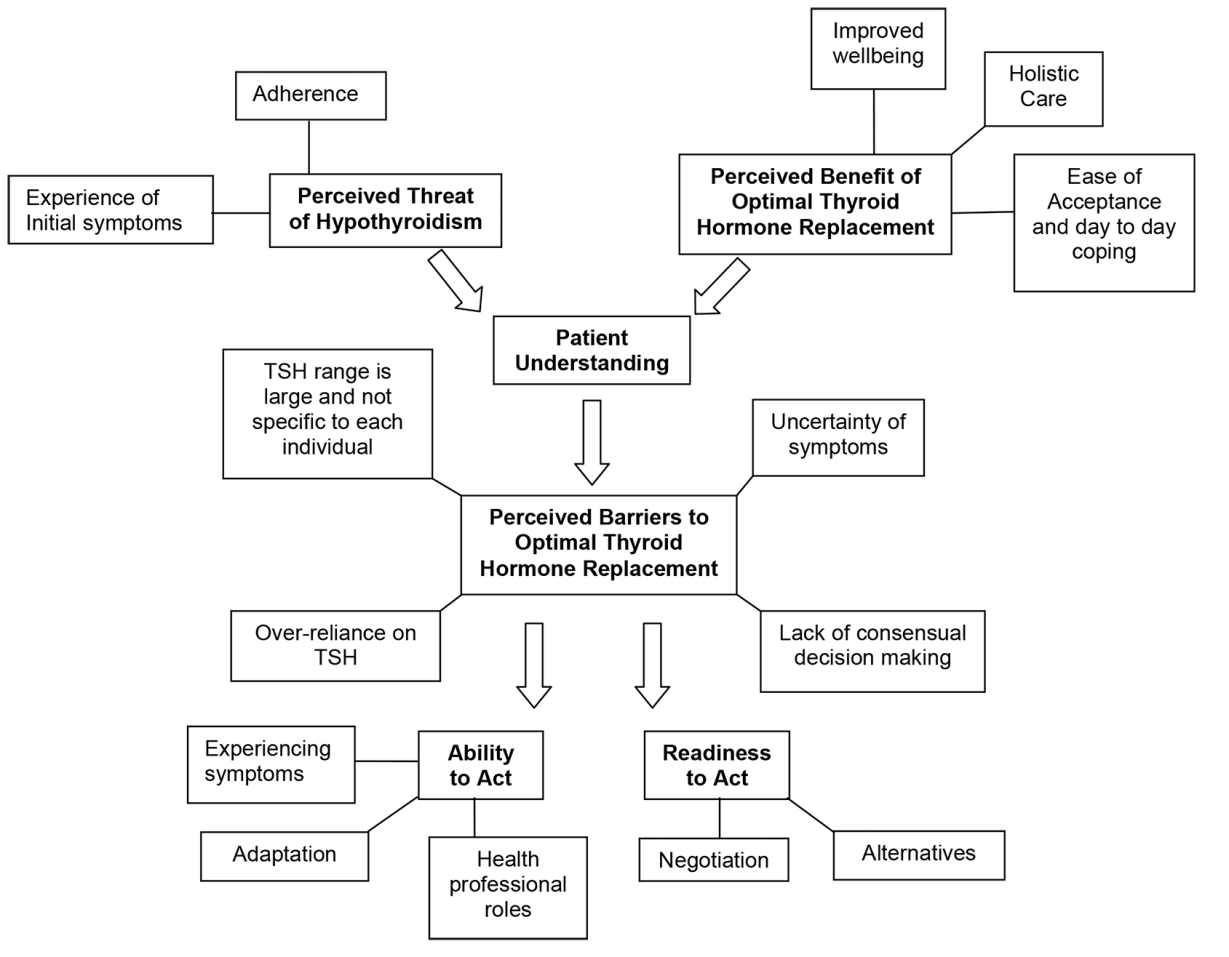
Concept diagram of categories and themes from interviews. TSH = thyroid stimulting hormone.

Data collection and analysis were conducted in parallel. Interview data were initially open coded and then analysed using constant comparison, allowing for generation or re-classification of themes. As the interviews progressed, themes emerging from the data informed questioning in subsequent interviews.^31^ Finally, selective coding was used to identify the core categories and themes.^27^ In-depth data analysis was conducted by two researchers and a third researcher was involved in the overall explanation of the data. Emergent themes were mapped to the constructs of the HBM (Figure 1).

## Results

### Perceived threat of hypothyroidism: susceptibility and severity

Patient perception of threat of disease was strongly linked to their experience of symptoms prior to diagnosis. Severe tiredness was the most commonly described symptom, while weight gain, dry skin, ‘brain fog’, and scalp hair loss were less common:

‘Well, it was at a point where I was just totally physically exhausted, you know. The 10-minute walk to work that I had at the time was taking every ounce of energy. That’s how I put it to the GP. And my brain was a fog as well. I couldn’t think straight at work.’ (Patient-22, male, 68 years old)

The patients that had not experienced symptoms prior to diagnosis were mostly unfamiliar with the typical symptoms of hypothyroidism and perception of disease susceptibility and severity was low:

‘So, in a sense, I suppose I’ve become positively confidently complacent about what I still don’t describe as a condition. As I didn’t have symptoms, other than the lumps which then led to the subtotal [thyroidectomy], which then led to the replacement by chemicals, which continues and hasn’t changed.’ (Pateint-23, male, 69 years old)

Perception of threat of disease was demonstrated by reported adherence to treatment, and patients felt they needed to take their levothyroxine to prevent the symptoms of hypothyroidism. Only one interviewee who had serum TSH levels within the reference range reported forgetting ther medication at weekends as they kept their levothyroxine at work, while one patient with out of range TSH regularly forgot to take their tablets as they were trying to avoid taking them with food:

‘Because I don’t want to be like I was before I didn’t have them [levothyroxine tablets]’. (Patient-24, female, 64 years old)

‘I don’t think I’ve ever … ever forgotten to take them [levothyroxine tablets].’ (Patient-14, female, 69 years old)

### Perceived benefits of optimal thyroid hormone replacement

Patients felt that their wellbeing and performance had improved with levothyroxine and the majority of patients felt well regardless of whether their TSH levels were in range or not:

‘I think, now that I’m on the levothyroxine, my life is a lot clearer, almost. You know, I haven’t got that fog, that lack of motivation and that tiredness hanging over me. So dealing with the condition now I’m medicated for it, to be fair, is a breeze compared to actually living with it when you don’t realise you’re living with it.’ (Patient-16, female, 37 years old)

Patients that felt well perceived that the personalised approach adopted by their GP had contributed to making them feel better. This included regular checks of their thyroid function tests and adjustment of levothyroxine doses to achieve the desired symptom control:

‘I get it checked probably more regularly than I’m meant to. And again, once I get it checked, it’s not the blood test they’ll look at —﻿ they’ll [GP] say, “How do you feel? You know, is it …? Do feel any better? Do you feel worse?” And they adjust it according to that.’ (Patient-12, female, 38 years old)

Generally, patients’ attitude to their disease was positive and they saw the benefit in taking levothyroxine. Patients felt they could live normal lives, and accepted that treatment was permanent:

‘It’s so important that you can look around other people who are struggling medically and you think, well, at least if you’ve got your health. If you’ve got the ability to get out there and enjoy life, that is so important.’ (Patient-11, male, 68 years old)

‘I just accept I’m going to take the tablets for the rest of my life. You know. It’s no big deal, really.’ (Patient-21, female, 60 years old)

### Patient understanding

Generally, patients had poor knowledge of thyroid function, the consequences of poor treatment adherence, or the risks associated with being out of the therapeutic treatment range, although a few patients were more informed than others. The perceived requirement for knowledge by a patient will have been influenced by the perceived threat of hypothyroidism, and the perceived benefit of optimal thyroid hormone replacement therapy:

‘I haven’t an idea. I haven’t any idea how the thyroid works.’ (Patient-20, male, 77 years old)

‘But I didn’t think it was a good idea to not be taking the right dosage. So … And I don’t know what would happen if I went in reverse and I didn’t take it [levothyroxine].’ (Patient-15, female, 49 years old)

‘Well, my best understanding is I think it [thyroid] produced four hormones, and at least one of them decays into another. And what they’re doing is topping up one of those hormones with this thyroxine. Which I believe takes 3 or 4 days to break down anyway. And also that your thyroid gland is really, really important in your metabolic rate and …’ (Patient-22, male, 68 years old)

A barrier to patient understanding was that most patients said that they had not been given information at the time of their diagnosis. Although some patients recall receiving a leaflet, generally, patients did not remember very much about it:

‘I think the key word I can think of is almost “hands-off”. Because obviously my diagnosis was via the phone. A lot of my blood tests have been, like, either organised or the results given via the phone. And very, very little face-to-face contact. And no … no information.’ (Patient-16, female, 37 years old)

Some patients described that they had done their own research in an attempt to gain information. However, poor patient knowledge was exacerbated by the complexity of the information presented, and patients therefore felt that researching on the internet could be inaccurate and could provoke anxiety, and were often put off researching:

‘Because I’ve spent so much time on Google, essentially. And half the time I don’t know if what I’m reading is … you know. If it’s … if it’s wholly accurate or not. If it’s … sometimes if it’s scaremongering or if it’s, you know, just people over exaggerating.’ (Patient-16, female, 37 years old)

The majority of patients with TSH levels outside the reference range were unaware of this. When followed up it was found that over half of these patients were being over-replaced (low TSH) (Table 1). Patients who are over-replaced may be asymptomatic and therefore unaware of their over-suppressed TSH level.

### Perceived barriers of optimal thyroid hormone replacement

Patients that had a normal TSH result but still felt unwell thought that their symptoms were being ignored by their GP or assumed them to be unrelated to their thyroid condition, and felt that their good TSH result presented a barrier to optimising their treatment. An empathetic and holistic approach as suggested by Patient-8 may prove more beneficial in identifying and resolving these symptoms rather than a singular focus on TSH as a measure of wellbeing:

‘I just feel that as long as you’re within the range, no matter how close to the cusp —﻿ as long as you’re within the range, they go “Just leave you at that.” They don’t look at the symptoms you’re having or the, you know … the battles that you’re having. You know?’ (Patient-4, female, 62 years old)

‘I think it’s … It could probably be monitored better by looking at the whole … At the patient. And when things are normal, I think we’ve got to remember that that’s just an average. And everyone is … What might be normal for me, might not be … you know, it’s different for everyone.’ (Patient-8, female, 63 years old)

Patients that felt unwell also believed that TSH levels were too crude a measure to gauge optimal thyroid hormone replacement. Some more informed patients had approached their GP and asked for further tests to check their triiodothyronine (T3) and thyroxine (T4) readings, as they felt unwell and dissatisfied with their treatment. However, since T3 measurements have limited value in the management of hypothyroidism,^32^ these tests are not routinely offered:

‘When I’ve mentioned previously about associated conditions that … And the T3, I was met with the fact that everything else looked normal —﻿ therefore there was no need to do other investigations. Which I find … I do find that quite sad, actually. Because, yes, then it does make you feel like you’re not cared enough about.’ (Patient-5, female, 47 years old)

Patients perceived that tiredness and memory problems could be due to aging, inadequate sleep, or from having a long day rather than specifically related to their hypothyroidism. Patients with symptoms such as cold extremities and dry skin felt that they had always had these problems and so were unsure if they could relate it directly to their thyroid. Lack of understanding of their condition and uncertainty regarding the cause of their symptoms may present as a barrier to further discussion with their GP to address these symptoms and improve wellbeing:

‘It’s like, you know, I’ve got an 8-month old. She still wakes up on her own schedule. And, like, so am I tired in the morning because she’s had a … like, she’s got up an extra time or just taken that little bit longer to settle. Or am I tired because it’s a thyroid? The thyroid is so difficult.’ (Patient-16, female, 37 years old)

The majority of patients felt their thyroid was the cause of their symptoms, however, two patients perceived that symptoms might be attributable to a health problem other than hypothyroidism. Moreover, after a thyroid function test, one of these patients felt her symptoms were a result of another health concern:

‘I’ve lately had a lot of symptoms, which led to me getting my thyroid re-checked and … which is … moving on to, like, looking at other causes. Where I have got a lot of, like, tiredness, headaches, stiffness in hands and feet. So I don’t believe it’s currently down to the thyroid.’ (Patient-16, female, 37 years old)

‘You can’t blame everything on the thyroid, you see. Because you could have something … your heart … or something like that.’ (Patient-27, male, 58 years old)

The GP or endocrinologist was generally seen as responsible for achieving adequate thyroid hormone replacement, rather than patients themselves. Some patients reported that they felt that there was a lack of consensual decision making between them and their GP, and this may present a barrier to optimising thyroid control if patients felt their GP could improve their treatment, but did not want to contradict their doctor. Moreover, some patients reported that they had not asked for advice or mentioned their symptoms as they did not want to ‘bother’ their GP:

‘It is my responsibility, I know that, for my health and wellbeing and everything. But it’s up to the doc, because it’s a tablet, isn’t it? It’s not taking two paracetamol. It’s up to the doctor to tell me what I should be doing. I don’t know if that’s right or not, but that’s what I feel anyway.’ (Patient-24, female, 64 years old)

‘Well, let me put it this way. I think if they’ve qualified to be a doctor, or a nurse, they … In my opinion, sometimes —﻿ not always, but sometimes —﻿ know better than me. Because they’ve got all the information at their fingertips. I haven’t.’ (Patient-19, female, 71 years old)

‘I’m not embarrassed about the fact that I’ve got this and got that. I’m not shy about that. But you just feel, oh … I’m bothering them.’ (Patient-4, female, 62 years old)

### Ability to act

Experience of symptoms was a cue to action seen from the data. Patients who were still experiencing symptoms, even those with a normal TSH result which was a perceived barrier to optimal thyroid control, felt disheartened and frustrated about their wellbeing but continued to take their levothyroxine as they felt it made some improvement:

‘It is. It is —﻿ it’s really frustrating because you think that some people lose weight so quickly. But, I mean, it’s taken me since January to get 2 stone off.’ (Patient-1, female, 36 years old)

Using a tablet box, keeping their levothyroxine in a visible or daily accessed place such as by the coffee, in their makeup bag, by their bed or next to their toothbrush, or having a family member reminding them were strategies that patients used to reduce the risk of non-adherence to levothyroxine and avoid suboptimal treatment:

‘They’re in a little bag in that chest of drawers. And that’s the first job every morning, to take my tablets and get my mascara on. It’s all in the same bag, so …’ (Patient-21, female, 60 years old)

Some patients reported receiving thyroid treatment promoting advice from their pharmacist such as regarding timing of medication, avoiding food and other medication. Additionally, most patients felt that if they needed help such as accidental overdosing or if they thought they needed their dose altering or were not feeling well they would ask their GP:

‘What he has done, and I think annually now — so just the last 2 years —﻿ when I’ve gone in, he says, “Do you have a few minutes, just to go over your medication?” So he [pharmacist] checks how I am.’ (Patient-23, male, 69 years old)

‘I know that I can go to the doctors and get the medication to control it.’ (Patient-2, female, 85 years old)

### Readiness to act

Few patients were unhappy to take levothyroxine every day, but were aware they needed to. One patient had negotiated with her GP to take 50 rather than 100 µg/day as she did not like medication and preferred homeopathy, demonstrating aspects of negotiating preferences with medication taking and shared decision making:

‘Really she [GP] would be happier if I took 100 µgs. And that was when I said, well, would it be dangerous if we keep it to 50? And she said no. And I said, “Well, I would like to keep it at 50”.’ (Patient-26, female, 68 years old)

Patients with TSH levels within range that reported feeling symptomatic had researched alternatives to levothyroxine, such as armour thyroid (desiccated porcine thyroid extract), combination therapy with liothyronine (synthetic T3), and homeopathic options in an attempt to find something to further relieve symptoms. However, patients did not recount speaking to their GP about an alternative, and one patient mentioned that she thought it was pointless to ask her GP about armour thyroid as her requests for T3 and T4 readings had previously been rejected, highlighting the lack of shared decision making that was previously identified as a barrier to optimal thyroid hormone replacement:

‘Yeah. And I thought it was pointless going to my doctor about that [armour thyroid].’ (Patient-8, female, 63 years old)

## Discussion

### Summary

Perception of disease threat was high in patients in this study that had experienced hypothyroid symptoms at diagnosis, and this was evident from reported excellent levothyroxine adherence. Improved wellbeing and performance, and the ability to cope with the condition were the perceived benefits of optimal thyroid hormone replacement. Poor patient knowledge of thyroid hormone replacement was likely due to lack of credible information, and was influenced by perceived threat of disease and assumed benefits. Barriers included lack of patients’ authority to challenge the GP, uncertainty regarding the significance of their symptoms, and over-reliance on TSH within the reference range as the sole measure of adequate thyroid hormone replacement. The ability to act was influenced by experience of symptoms and shown by strategies to promote drug adherence, while patient readiness to act was demonstrated by negotiation of their medication preferences and/or seeking alternatives to levothyroxine in patients that did not feel well replaced.

### Strengths and limitations

By using in-depth discussions with patients in the absence of any healthcare professional it was possible to gather rich data of the personal thoughts and feelings of patients with hypothyroidism. However, it is possible that the perspectives of the researcher led to bias, influencing how the patients responded to the questions in the interviews or data analysis. As the interviewer did not have a clinical background or strong prior assumptions, it is unlikely that interviewer bias would have influenced these results.

The participants were sampled from 11 GP practices in a single locality in the UK and therefore caution about generalising these findings to other populations and healthcare settings is needed. Over half of patients with suboptimal thyroid hormone replacement had TSH values lower than the reference range. Interviewing more patients with high TSH may have given different outcomes. However, the authors aimed to study a broad sample of patients with hypothyroidism including well-controlled, overtreated, and undertreated patients, in order to obtain insights across a range of thyroid statuses. As the results demonstrate, some patients with suboptimal thyroid hormone replacement were satisfied with their treatment while other patients with normal TSH remained unwell.

### Comparisons with existing literature

Similar to the current study, in previous qualitative research, patients with hypothyroidism described tiredness as the most common symptom they had experienced, and lack of information exchanged between patient and doctor was also acknowledged.^33^

Additionally, the vague nature of hypothyroid symptoms often lead to dissatisfaction in patients and symptoms being discounted or blamed on other causes by patients and health professionals.^33,34^

Studies have demonstrated that enhanced knowledge about disease can lead to a higher perceived involvement in medical decisions,^35,36^ and may be highly influential in improving compliance.^36^ Lack of shared decision making (SDM) was a barrier identified in the current study. In a qualitative study by Britten *et al*,^37^ a lack of SDM in GP consultations was found to be influenced by patients not voicing agenda items, aversion to taking medicine or requests for medication, and misunderstandings were seen by both patient and GP. Moreover, in instances where patients did voice their concerns, this was often unexplored by their doctor. With this in mind, increasing SDM in patients with hypothyroidism may help optimise treatment, similar to that in patients with hypertension where improved blood pressure control and adherence was seen in patients involved in SDM.^38^

Previous studies using questionnaires have found a reduction in neurocognitive functioning, psychological wellbeing, and quality of life in levothyroxine treated patients with normal TSH levels compared to population reference standards^39^ or age- and sex-matched controls.^40^ The current study thus highlights the barriers faced by such patients in achieving wellbeing, including uncertainty regarding the significance of their symptoms, fear of challenging their GP’s attitude, and the over-reliance on biochemical indices by health professionals.

Patients who have persistent symptoms despite a TSH concentration within the reference range should be carefully evaluated for other causes of their symptoms including a range of comorbidities and lifestyle factors.^41^ Surks *et al* acknowledged that GPs should have an individualist approach to each patient as the TSH reference range applies to populations,^42^ supporting over-reliance on TSH levels as a barrier to optimal thyroid replacement for each patient. Additionally, previous research has shown that some patients feel better being slightly over-treated,^43^ although persistently very low or fully suppressed TSH levels (≤0.03 mU/L) can increase the risk of cardiovascular events and fractures.^11^

Other health belief studies have found a strong link between high perceived threat of chronic disease and good patient medication adherence.^44,45^ For hypothyroidism, non-adherence to levothyroxine therapy has been reported to be as low as 22%,^46^ but the traditional belief that poor adherence causes suboptimal thyroid treatment was not supported by this study.

### Implications for future research and clinical practice

Although levothyroxine therapy will return TSH levels to within the normal range, this range is wide and it can be difficult to ascertain an individual’s optimal TSH set-point. Thus the use of TSH reference range and the role of symptom control require further investigation. However, the cost-effectiveness of these strategies and the cardiovascular and osteoporosis risks that are associated with very low TSH levels should be considered.

Implementing the provision of standardised information at the time of diagnosis such as leaflets from patient.co.uk, which this study's cohort reported not receiving, having a medication review with their pharmacist, or directing patients to patient support groups, such as the British Thyroid Foundation, may help to improve patient knowledge and understanding, reduce uncertainty of symptoms and promote GP and patient SDM. However, use of an educational booklet during a randomised controlled trial showed no benefits on levothyroxine adherence or wellbeing in patients with hypothyroidism.^47^ Thus, whether benefits from educational interventions can be achieved within routine practice will require further research. Structured education has proved successful in other chronic disease models, such as diabetes mellitus, and similar models could be adapted in patients with hypothyroidism.

Investigating the perceptions of health professionals involved in the management of hypothyroidism is crucial, particularly the responsiveness to patient’s symptoms, over-reliance upon TSH levels, and the community pharmacist’s role. Focusing on the synergy of patient concerns, the dose and time of administration of levothyroxine, the dietary advice given, and the role of the GP have been described to be key to ensuring the appropriate therapeutic outcome of levothyroxine.^48^ Following on from this, systematically quantifying the effect of concomitant medication, timing of administration and interference from diet on the bioavailability of levothyroxine may further inform hypothyroid treatment strategies. Lastly, there is now a growing interest in the effect of pharmacogenomic factors on wellbeing in patients with hypothyroidism. Common genetic variations in the DIO2 deiodinase gene involved in thyroid hormone action have been linked with impaired psychological wellbeing in subgroups of patients on levothyroxine, but further studies are required to confirm this association and whether alternative treatment with liothyronine will benefit such groups of patients.^49^
